# Generation of Reactive Oxygen Species (ROS) and Pro-Inflammatory Signaling in Human Brain Cells in Primary Culture

**DOI:** 10.4172/2161-0460.S2-0011

**Published:** 2012-01-25

**Authors:** Walter J. Lukiw, Surjyadipta Bjattacharjee, Yuhai Zhao, Aileen I. Pogue, Maire E. Percy

**Affiliations:** 1 LSU Neuroscience Center and Department of Ophthalmology, Louisiana State University Health Sciences Center, New Orleans LA 70112, USA; 2 University of Texas Health Science Center, Houston TX 77030, USA; 3 Alchem Biotek Corporation, Toronto ON M5T 1L8, Canada; 4 Surrey Place Centre, Toronto ON M5S 2C2 and University of Toronto Departments of Physiology and Obstetrics & Gynaecology, Toronto ON M5S 1A8, Canada

**Keywords:** Alzheimer’s disease, Apoptosis, Fenton chemistry, Human neural cells, Inflammation, Metal sulfates, Synergistic effects

## Abstract

The cellular generation of reactive oxygen species (ROS) has been implicated in contributing to the pathology of human neurological disorders including Alzheimer’s disease (AD) and Parkinson’s disease (PD). To further understand the triggering and participation of ROS-generating species to pro-inflammatory and pathological signaling in human brain cells, in these experiments we studied the effects of 22 different substances (including various common drugs, interleukins, amyloid precursor protein, amyloid peptides and trace metals) at nanomolar concentrations, in a highly sensitive human neuronal-glial (HNG) cell primary co-culture assay. The evolution of ROS was assayed using the cell-permeate fluorescent indicator 2’,7’-dichlorofluorescein diacetate (H_2_DCFDA), that reacts with major ROS species, including singlet oxygen, hydroxyl radicals or superoxides (λEx 488 nm; λEm 530 nm). Western analysis was performed for cyclooxygenase-1 (COX-1), cyclooxygenase-2 (COX-2) and cytosolic phospholipase A (cPLA_2_) to study the effects of induced ROS on inflammatory gene expression within the same brain cell sample. The data indicate that apart from acetylsalicylic acid (aspirin) and simvastatin, several neurophysiologically-relevant concentrations of Aβpeptides and neurotoxic trace metals variably induced ROS induction, COX-2 and cPLA_2_ expression. These findings have mechanistic implications for ROS-triggered inflammatory gene expression programs that may contribute to AD and PD neuropathologic mechanisms.

## Introduction

Age-related neurological disorders such as Alzheimer’s disease (AD) and Parkinson’s disease (PD) have long been associated with free radical-induced oxidative stress, and these are driven by the non-homeostatic production of reactive oxygen species (ROS) [1–8]. ROS are highly reactive and charged metabolic intermediates that attack DNA, RNA, protein and lipids to leave oxidized cellular components that are genotoxic or cytotoxic, and unable to perform their normal biological functions. While cellular systems have evolved elaborate anti-oxidant systems to neutralize the effects of ROS, degenerative disease processes such as those associated with AD and PD may overwhelm these neuroprotective anti-oxidant defenses [5–11]. The progressive generation of ROS during the course of human aging lies at the core of the free radical theory of aging originally proposed by Harman 55 years ago [10]; this theory implies that aging is associated with increased ambient levels of ROS, ROS-oxidized biomolecules and their deleterious biological effects [8–11].

Based on a previously verified and highly sensitive assay in vitro stress-test system for the effects of ROS on human brain cell pathogenic gene expression [9,12–18], in these experiments we tested the ROS-inducing effects of the two most widely used non-prescription drugs – simvastatin and acetylsalicylic acid (aspirin); the pro-inflammatory cytokines – interleukin-6 (IL-6) and interleukin-1beta (IL-1β); tissue necrosis factor alpha (TNFα); amyloid precursor protein (βAPP) and the AD-associated neurotoxic peptides – Aβ40 and Aβ42; the neurotoxic metals – Hg, Cu, Zn, Mn, Fe and Al; and hydrogen peroxide at 50 nM or 100 nM ambient concentrations in the growth medium of human neuronal-glial (HNG) cells in primary culture. Several of these factors were tested in combination. Because brain cells sense applied physiological stress as a form of impending cellular injury, they respond through an up-regulation of immune and inflammatory gene expression which is, in part, neuroprotective [19,20]. However excessive stimulation of these immune and inflammatory pathways are highly detrimental to normal brain cell with the induction of stress-responsive, pro-inflammatory and pro-apoptotic gene expression programs that may initiate, enhance and/or accelerate brain cell decline [18–20].

## Materials and Methods

### Reagents and antibodies

Simvastatin (1S,3R,7S,8S,8aR)-8-{2-[(2R,4R)-4-hydroxy-6-oxotetrahydro-2H-pyran-2-yl]ethyl} -3,7-dimethyl-1,2,3,7,8,8a-hexahydronaphthalen-1-yl2,2-dimethylbutanoate; S6196) and acetylsalicylic acid (O-acetylsalicylic acid, ASA, aspirin; A5376) were dissolved in DMSO and ultrapure water, respectively, following instructions provided by the manufacturer (Sigma-Aldrich Chemical, St. Louis, MO). When appropriate, control HNG cells received DMSO at concentrations used in simvastatin and ASA assays. Solutions of βAPP, Aβ40 and Aβ42 were prepared as previously described [21–23]. All trace metals were used as ultrapure sulfates [14]. Briefly, Biochemika MicroSelect© ultrapure reagents for molecular biology, including MgSO_4_ (63133), Mn(II) sulfate (31425), Hg(II)SO_4_ (83372), Cu(II)SO_4_ (35185), ZnSO_4_ (35392), FeSO_4_ (44970) and Al_2_(SO_4_)_3_ (11044; Sigma-Aldrich or Fluka Chemical, Milwaukee, WI), freshly prepared as 0.1 M stock solutions [9,16,17], were instilled into serum-containing HNG cell maintenance medium (HNGMM, pH 7.5; see section below for details) by gentle inversion, followed by filter sterilization using 0.2-µM spin filters (Millipore Corporation, Billerica, MA). Solutions were used at the concentrations shown in Table 1. HNG cells, HNGMM and bullet packs containing human epidermal and fibroblast growth factor (E/FGF), gentamicin/amphotericin (G/A1000), neural survival factor-1 (NSF-1) and FBS were obtained from Clonetics-Lonza (Walkersville, MD). Western immunoblots were performed using human-specific primary antibodies against a beta-tubulin III (βTubIII T8660), glial fibrillary acidic protein (GFAP, G9269; Sigma-Aldrich Chemical). Antibodies to the control cytoskeletal filament β-actin (sc-81178), cyclooxygenase-1 (COX-1; sc-1752), cyclooxygenase-2 (COX-2; sc-1747) and cytosolic phospholipase A_2_ (cPLA_2_; sc-137089) were obtained from Santa Cruz Biotechnologies (Santa Cruz, CA). Hoechst 33258 bis-benzimide (H-1398), 2’,7’-dichlorofluorescein diacetate (H_2_DCFDA; D399) was obtained from Molecular Probes-Invitrogen (Eugene, OR) and used according to the manufacturer’s instructions. All other reagents were of the highest ultrapure grades commercially available and were used without further purification [18–20].

### Human neuronal-glial (HNG) cells in primary culture

**Figure 1:**
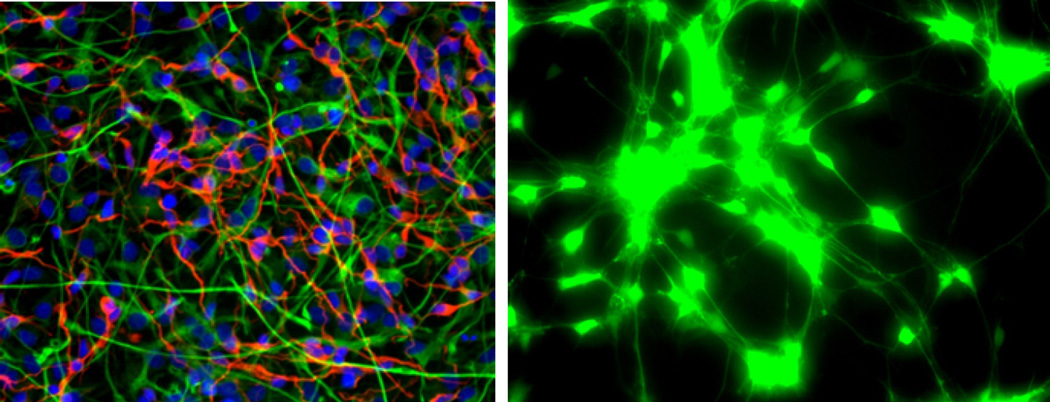
Human neuronal-glial (HNG) cells in primary culture **(A, Left panel)** two weeks in culture; approximately 50-50 neurons and astroglia; neuronal cells are stained with neuron-specific β-tubulin (red; λ_max_=690 nm), glial cells are stained with glial-specific glial fibrillary acidic protein (GFAP; green; λ_max_=525 nm), and nuclei are stained with Hoechst 33258 (blue; λ_max_=470 nm); magnification 20×; **(B, Right panel)** co-incubation with H_2_DCFDA indicates ROS generation; treatment after 3 hrs with Al+Fe (50 nM each, as sulfates) shown; green fluorescence (emission λ_max_=530 nm).

HNG cell lines, derived from normal human neural progenitor cells (PT-2599; Lonza Clonetics Cell Systems, Walkersville, MD) were cultured in 6-well (3.5 cm diameter) plates (Costar 3506, Corning Life Sciences, Acton, MA) at 5% CO_2_, 20% O_2_ and 37°C in an HNG cell maintenance medium (HNGMM) supplemented with 2.5% serum containing hFGF (human fibroblast growth factor), NSF-1 (neuronal survival factor 1), hEGF (human epidermal growth factor) and GA-1000 (gentamicin-amphotericin B G/A 1000) as previously described [9,12–19]. HNGMM was completely changed every 3.5 days. At 2 weeks of growth there were approximately 50% neurons and 50% astroglia (Figure 1). HNG cells tested positive for the neuronal- and glial-specific markers βTUBIII and GFAP, respectively, and tested negative for fibroblast contamination using antibodies against fibroblast-specific protein-1 (data not shown).

### Minimization of extraneous contamination

Throughout these experiments ultrapure water (18 megohm, Milli-Q, Millipore or Puriss 95305, Fluka) was employed in all cell culture, protein isolation and biochemical procedures to stringently exclude trace metal extraneous contamination; as analyzed by electro thermal atomic absorption spectroscopy, aluminum, copper, magnesium, mercury, iron and zinc content were <5 ppb. Coded isolation reagent and media samples were analyzed for potential trace metal contamination using a Perkin Elmer 5000PC Zeeman-type electro thermal atomic absorbance (EAA) spectrophotometer equipped with an automated sampler and IBM/AT-supported analysis package for trace metal analysis [9,18,19]. Wherever possible, ultrapure HNO_3_ washed polysulfone plasticware was used according to the URI-GSO protocols to stringently eliminate trace metal contamination [17–19].

### Western analysis and immunostaining

To ascertain whether up-regulation in specific protein species was associated with ROS production, Western immunoblots were performed using human-specific primary monoclonal antibodies to COX-1, COX-2 and cPLA_2_ using the β-actin protein signal in the same sample as control [14–19]. Bound primary antibodies were detected with an anti-IgG fluor-linked secondary antibody (PA45007; Amersham Biosciences, Piscataway, NJ) and developed with an ECL+ Western blotting system (RPN2132; Amersham).

### ROS Assay using 2’,7’-dichlorofluorescein diacetate (H_2_D-CFDA)

**Figure 2:**
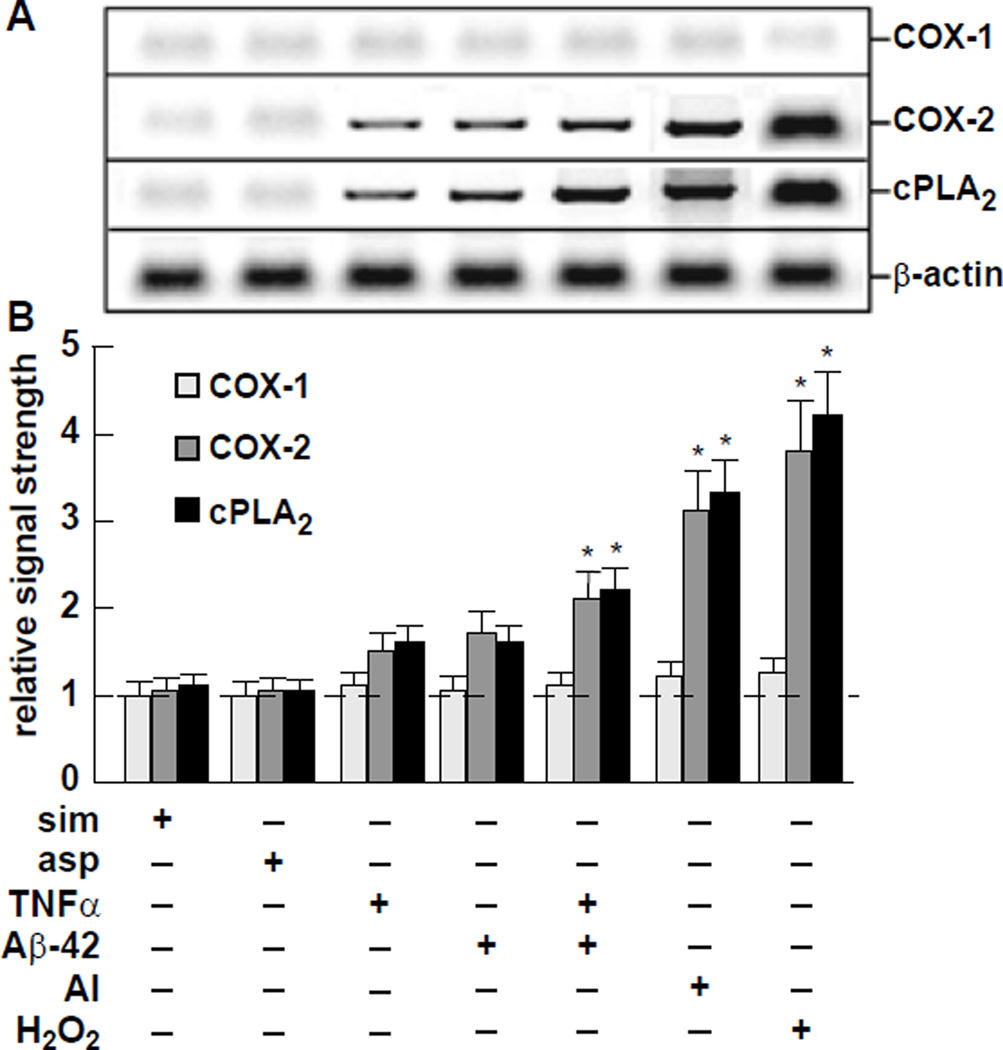
Western analysis of COX-1, COX-2 and cPLA_2_ in stressed HNG cells Typical results of Western analysis for COX-1, COX-2, cPLA_2_ and β-actin protein in HNG cells stressed with various drugs, neurotoxic amyloid peptides and trace metals, at nanomolar concentrations (*see also* Table 1); protein bands are shown in (A) and these results are quantified in bar graph format in (B). The relative ROS and Western signal strengths for all factors tested are represented in Table 1. For ease of comparison, a dashed horizontal line at 1.0 indicates relative signal strength for simvastatin and aspirin effects on the COX-1 signal; *p<0.05 (ANOVA).

Levels of reactive oxygen species (ROS) were assayed in metalion-treated and un-treated 2 wk old HNG cells (Figure 2) using 2’,7’-dichlorodihydro-fluorescein diacetate (H_2_DCFDA) at a 10µM ambient concentration in cell culture medium in the dark using protocols provided by the manufacturer (Molecular Probes). H_2_DCFDA, cell-permeate indicators that react with the highly reactive singlet oxygen, hydroxyl radicals or superoxide-generating fluorescent signals (collectively termed ROS), were quantified using electronic imaging photography under UV light (λEx 488 nm; λEm 530 nm) using a Zeiss Axioskop/Zeiss MC63 photo control unit coupled to a Nikon Optiphot 2 microscope equipped with an additional differential Interference Contrast/Nikon UFX DX photo control unit [9].

### Western analysis data and statistical analysis

Western signal-intensity data were gathered by phosphor imaging onto molecular imaging screens using a Typhoon (Amersham-Pharmacia Biosciences) Molecular Imaging system. All statistical procedures were carried out using the programs and procedures in the SAS language (Statistical Analysis System, SAS Institute, Cary, NC). All *p* values were derived from protected *t*-tests or least square means from a two-way factorial analysis of variance (*p*, ANOVA); only *p*-values of less than 0.05 were considered to be statistically significant.

## Results

A typical culture of HNG cells used in these experiments is shown in Figure1A, and a typical H_2_DCFDA-based ROS assay is shown in Figure 1B. The example shown in Figure 1B is from the intense degree of ROS generated in HNG cells stressed with Al+Fe (50 nM each, as sulfates). Fluorescent signals from stressed HNG cells were quantified using digital electronic imaging photography under ultraviolet (UV) light (λEx 488 nm; λEm 530 nm) employing an Axioskop/Zeiss MC63 photo control unit and a Nikon Optiphot 2 microscope equipped with an additional differential-Interference Contrast/Nikon UFX DX photo control unit.

The ROS signal intensity for 22 test compounds including Aβ40- and Aβ42peptides and trace metals are shown in Table 1; depending on ROS generated a scale from 1–10 was derived from the ROS signal obtained from control HNG cells as previously described [9,18]. HNG cells treated with MgSO_4_, simvastatin, aspirin or βAPP at any concentration tested for 3 hrs showed no generation of ROS above control values. Aβ40 peptide at 10, 50 and 100 nM ambient concentration showed relatively modest ROS generation; Aβ42 peptide at 10, 50 and 100 nM ambient concentration showed 3- to 5-fold the ROS induciblity as did Aβ40 peptide. Aβ40 and Aβ42 peptide together showed no significant synergistic effects (data not shown). Interleukin-6 (IL-6), interleukin 1-beta (IL-1β) and tissue necrosis factor alpha (TNFα) showed greater induction of ROS than Aβ40 or Aβ42 peptides alone. Interestingly the combination of Aβ42+IL-6 showed no synergistic effect of ROS generation while the combinations of Aβ42+IL-1β and Aβ42+TNFα showed a higher ROS generation than Aβ42, IL-1β or TNFα alone.

Concerning the ability of metal sulfates to generate ROS the order of effectiveness was Al>>Fe>Zn>Mn>Cu>Hg. The combination of Al+Fe (as sulfates) and H_2_O_2_ by itself showed the greatest ability to generate ROS at the concentrations tested.

Using Western assay the levels of COX-1, COX-2 and cPLA_2_ were also analyzed in these samples, and we observe a significant correlation between ROS generation and COX-2 and cPLA_2_ up-regulation; no significant induction was observed in the control COX-1. For example, the greatest inducers of ROS identified in this study, Al+Fe (as sulfates) and H_2_O_2_ by itself, associated with the highest induction of both COX-2 and cPLA_2_, each to at least 3-fold or greater over controls.

## Discussion

The original experimental design and aim of this study was to quantify the relative ROS-generating capability (and ensuing genetic toxicity) of several physiologically-relevant neurotoxic factors using human neuronal-glial (HNG) cells in primary co-culture. These primary cell cultures provide the basis for a highly sensitive and proven primary brain cell analytical assay that is representative of all human neocortical brain cell types. Although the present study was limited to study of ROS formation and activity of COX-1, COX-2 and cPLA_2_ levels, the ROS-induced expression of COX-2 and cPLA_2_ (but not COX-1) by different neurotoxins is by itself highly indicative of ROS-mediated activation of the arachidonic acid cycle in primary HNG cells.

Excessive ROS generation in brain cells and CNS tissues promotes cellular oxidative stress that progressively renders normally functioning DNA, RNA, proteins and lipids incapable of performing their normal cellular functions: this is the basis of the free-radical theory of aging [1,4,5,10,11]. Aging is the greatest known risk factor for the onset of neurodegenerative diseases such as AD and PD, progressive, incurable neurological disorders with a prevalence of 67 AD and 10 PD cases per 1000 elderly persons [24]. In the aging brain, mitochondrial dysfunction increases with age, and increased production of ROS and oxidative stress is highly damaging to both neurons and glia in these common human neurodegenerative conditions [4,5,11,20]. The use of anti-oxidants and free radical trapping agents have shown significant benefit in reducing oxidative stress and ROS generation in these in vitro test systems and in human clinical trials [15,17,25,26].

Simvastatin, a generic statin that lowers cholesterol levels through inhibitory actions on HMG-CoA (3-hydroxy-3-methylglutaryl-CoA) reductase, and aspirin, an non-steroidal anti-inflammatory drug that functions as a COX-1 and COX-2 inhibitor, are respectively, the two most widely prescribed and over-the-counter used drugs today in industrialized societies [27–29]. Both showed no significant promotion of ROS activity in the HNG cell assay system used here. Table 1 shows the relative degree of the induction of ROS for 20 other brain-relevant Aβ peptides, cytokines, toxic metals, and the synergistic effects of some of their combinations at 50 nM and 100 nM ambient concentrations in the HNG tissue culture medium. While the most potent single amyloid peptide inducer of ROS was Aβ42 peptide and the most potent cytokine inducer of ROS was TNFα, the combination of Aβ42+TNFα showed a synergistic enhancement in the production of ROS. The most potent single metal sulfate inducer of ROS was Al (as sulfate), and the most potent combinatorial metal sulfate inducer of ROS was Al+Fe that compared to an equivalent ROS-inducing capability by hydrogen peroxide (H_2_O_2_) by itself.

These findings underscore the idea that neurotoxic amyloid peptides and trace metals, at physiologically realistic nanomolar concentrations and either alone of in combination, are highly effective in inducing ROS and pathogenic and pro-inflammatory gene expression programs [9,15–19]. Interestingly, Mn induced ROS generation and neurological deficits have been recently implicated in the neuropathogenesis of idiopathic Parkinson’s disease (IPD), amyotrophic lateral sclerosis (ALS), Alzheimer’s disease (AD) and prion disease [30]. Highly complex mixtures of Aβ40, Aβ42, cytokines and neurotoxic metals (as might be expected in vivo) are likely to induce synergistic effects in promoting stress and neurodegeneration [14–16]; unpublished observations. While the ‘housekeeping cyclooxygenase’ COX-1 was not found to be induced by any treatment in these studies, COX-2 and cPLA_2_ were found to be significantly up-regulated in ROS-stressed HNG cells. This may be particularly significant for pro-inflammatory signaling as COX-2 and cPLA_2_ represent the initial and rate-limiting enzymes in the arachidonic acid cycle, and pathogenic prostaglandin signaling pathways. In addition, ROS is a potent inducer of the pro-inflammatory transcription factor NF-κB that is up-regulated in both AD and PD, and both COX-2 and cPLA_2_ are at least under partial NF-κB-mediated regulation [31–33]. Highly specific chelators and innovative chelation strategies may be useful to neutralize the effects of neurotoxic metals in human brain cells [17,25,26]. Lastly, the HNG cell system described here may also serve as a suitable test platform to compare the effects of other physiologically relevant stressors and chelators, and other inhibitory molecules that may be useful in quenching ROS-induced pro-inflammatory signaling that contribute to neuropathogenic events.

Studies involving of human neuronal-glial (HNG) cells in primary co-culture carried out over the past 20 years have proved highly informative in neurodegenerative disease mechanistic studies [21,34–36]. These and the present study should encourage other researchers to experiment with the organic and inorganic neurotoxic factors utilized and characterized in this study, and further explore their effects in HNG cells and other more specific human neural cell systems. A major challenge in neurodegenerative disorders is the connection between intracellular protein inclusions, which are different for different neurodegenerative diseases, neuroinflammation, and the functional loss of neurons and synapses [35–38]. Laboratories that have access to fresh human brain tissue, might consider co-culturing dopaminergic, midbrain, or hippocampal cells (mostly affected in IPD and AD, respectively) in parallel with astrocytic or glial cells in order to explore the relation between exposure to ROS-inducing factors and formation of intracellular inclusions. To also consider is the fact that it now is possible to induce functional dopaminergic neurons from human embryonic stem cells and pluripotent adult stem cells [38]. Although the use of human cells is likely to shed better light on the origin and pathogenesis of AD, IPD and other human neurodegenerative disorders than rodent cells, co-culture of human cells will benefit from recent advances in glial-neuronal sandwich co-culture systems [39–41].

## Figures and Tables

**Table 1:** Effects of different stressors, at nM concentrations, on reactive oxygen species (ROS) generation in human primary neuronal-glial (HNG) cell cultures, and induced inflammatory consequences as indicated by COX-2 and cPLA2 up-regulation*.

physiological stressor	concentration	degree of induction of ROS**	COX-1***	COX-2***	cPLA_2_****	references^t^
simvastatin	50 nM	0	−	−	−	25,26
aspirin	50 nM	0	−	−	−	27
βAPP	100 nM	0	−	−	−	13,14,20
Aβ40	10 nM	1	−	−	−	13,14
Aβ40	50 nM	1	−	−	−	13,14
Aβ40	100 nM	2	−	−/+	−/+	13,14
Aβ42	10 nM	3	−	+	−	13,14
Aβ42	50 nM	4	−	+	−/+	13,14
Aβ42	100 nM	5	−	++	+	13,14
IL-6	50 nM	5	−	−/+	−/+	32
IL-1β	50 nM	6	−	+	+	28,33
TNFα	50 nM	6	−	+	+	35
Aβ42+IL-1β	50 nM (each)	7	−	+	+	33,36
Aβ42+TNFα	50 nM (each)	7.5	−	+	+	31,35
Hg	50 nM	1.5	−	−	−	16,36,39
Cu	50 nM	3	−	+	−	16,36,40
Zn	50 nM	4	−	+	+	16,36,41
Mn	50 nM	4.5	−	−/+	+	16,30,36
Fe	50 nM	5	−	−/+	+	9,16,31,36,42
Al	50 nM	9	−	++	++	9,16–19,31,36,42
Al+Fe	50 nM (each)	10	+	+++	+++	9,16–19,31,36
H_2_O_2_	50 nM	10	+	+++	+++	16,31,33

* Depends on age of cell cultures and cell type; HNG cells cultured for 2 weeks under optimum growth conditions; each physiological stressor was assayed three times.

** scale of 1–10 based on these 20 evaluations

*** detection of cycloxygenase-1 (COX-1), cycloxygenase-2 (COX-2) after 3 hrs treatment

**** detection of cytoplasmic phospholipase A_2_ (cPLA_2_) after 3 hrs treatment

t References quoted here include relevant research on metal-sulfate induced oxidative stress and inflammatory gene expression.
